# Picric Acid—Ophthalmia; Natr. Mur.—Neuralgia and Cough; Mang. Carb.—Colic; Spongia—Chronic Winter Cough

**Published:** 1881-08

**Authors:** E. W. Berridge

**Affiliations:** London


### CLINICAL BUREAU.

### CLINICAL CASES.

*Picric Acid*—Ophthalmia; *Nat. m.*—Neuralgia and Cough. *Mang. carb.*—Colic; *Spongia*—Chronic Winter Cough.

E. W. BERRIDGE, M. D., LONDON.

*Picric Acid in Ophthalmia.*—March 6th, 1879; Mr.——, æt. 21. Intense pain in right eye since yesterday afternoon, as if something were in the eye; right eyeball and conjunctiva palpebralis red; the eye waters much, and the sight is dim; when reading, the letters seem confused; the pain relieved by cold bathing.

*Diagnosis of remedy:* Under *Picric acid* we find "conjunctivæ greatly inflamed, *right eye is the worst,* hard work to keep eyes open; makes eye feel sticky to read; *eyes better from washing in cold water,* and in cold air; worse in warm room." The only remedy which has the sympton of the *right* eye relieved by cold washing is *Thuya,* but the particular symptom thus relieved is *heat* (see my Eye Repertory, pp. 291, 294), whereas the *Picric acid* symptom points to a severe pain. I gave the patient in the morning one dose of *Picric acid,* CM. (Swan). The same evening he was better; there was further improvement next day, and he soon recovered. On April 12th he had remained cured.

*Nat. mur. in Neuralgia and Cough.*—February 10th, 1868. Mr.——, allopathic chemist, has had for about a week a feeling of fullness across right supra-orbital ridge, as if it were being pushed out from within, gradually getting worse; to-day it has extended to the corresponding part of left side; soreness on pressure on under surface of right supra-orbital ridge, in the bone, with feeling of warmth there. The fullness was worse to-day after bending the head down for sometime. It came on this morning in bed, from 4.30 to 6.30 o'clock, but only on right side, preventing sleep; he fell asleep at 6.30 o'clock, and was free from pain; the pain returned about 10 A. M., forty-five minutes after breakfast.

The right eye feels as if it would water; right upper lid hangs down lower than left, and is rather red, it feels warm. Feeling as if something were repeatedly pressing on under surface of right supra-orbital ridge, near inner canthus; he describes it by placing a finger on his hand, and repeatedly pressing it in; he distinctly says it is not a throbbing pain. To-day the pain was very bad at dinner, better in afternoon; the pain has generally been worse in evening, on sitting down to read. Three weeks ago caught cold; two weeks ago cough came on, excited by tickling at throat-pit. Now has cough excited by a tickling behind sternum, running up to throat-pit; it is worse in evening when sitting down to read; it comes on also when beginning to eat, especially if he drinks nothing; it is the same in and out of doors. For the last three days (this is the latest symptom) the cough has caused a feeling of a rush of blood from nape into occiput, compelling him to place his hand on occiput, which relieves it; from the first, though not every day, it has caused a pain from between shoulders through to chest; there is slight expectoration in the morning from the cough. For some months has had a feeling of stiffness of the nape on waking, as if he had lain on his back in the form of an arch, going off soon after rising. For many years has had fullness of vertex and forehead on waking. He eats salt with his food, though he dislikes it, because he has understood it is good to eat it! Three years ago was vaccinated for the second time; since then his teeth, which were perfectly sound before, *have begun to decay close to the gums.* (This is Bœnninghausen's characteristic of sycotic teeth; I have found it a valuable indication in toothache for *Thuya*). He has taken two doses of Pyretic salts, and during the first week of the cough applied mustard plasters, but all without effect.

*Diagnosis of the remedy*: The *totality* of the symptoms could not be covered. The most *peculiar*, and, at the same time, the *latest* symptom was the rush of blood to the occiput when coughing, relieved by pressure; this symptom, therefore, was, according to

HAHNEMANN, of the greatest importance. *Nat. mur.* has a very similar symptom, "Headache from sneezing and coughing, immediately disappearing on external compression;" it has also tenderness of left orbital region; it is also, in a high potency, an antidote to the effects of crude salt, which I considered to be the cause of his symptoms. Some persons are very sensitive to salt, and suffer from its pathogenetic action if they take it with their food. It is an error to suppose that raw salt is a necessary article of food because it exists in the tissues; iron and phosphorus also exist in the tissues, yet we are not taught to take these substances as we do salt; the truth is that food, in its natural state, as well as the air we breathe, contains sufficient of all these mineral substances to supply the waste continually going on in the body; when they are thus taken, in what we may call their organic form, they act as foods; when taken raw, as poisons or medicines, as the case may be. The common argument that animals eat it is fallacious, as they only take it occasionally, doubtless through an instinctive craving for the remedy for their morbid condition. The arguments derived from the condition of prisoners fed on unsalted food is also deceptive, as other causes were operative. Healthy children never, I believe, eat salt till the taste is acquired through compulsion; but I have frequently noticed a craving for salt associated with serious disease.

It is now more than twelve years since I myself gave up the use of raw salt, and I have not yet suffered the fate of Herod, as a kind friend predicted at the time.

*February 11th.* Woke with the same fullness and pain across eyebrows, mostly on right side; did not feel the soreness; lay awake from 5 to 6 A. M. At 8.20 A. M. took one globule of *Nat. mur.* 1000 (Jenichen); he was also instructed to leave off the use of salt entirely, and coffee while under treatment. This last advice I now consider to have been unnecessary; high potencies are not easily, if at all, disturbed by crude drugs, to which the patient is habituated. After the dose, felt extremely tired, low spirited, out of sorts entirely (effects of *Nat. mur.?*) Head became decidedly better; the usual evening aggravation did not occur. During and after dinner, the pain over eye was bad, but less than at the corresponding time yesterday. Cough much better, still preceded by the tickling; only once or twice felt the rush of blood to the head; no pain from back to chest. The pain, like repeated pressure in orbit, is almost gone. Cough did not come on at dinner or tea, and only slightly at supper. No feeling of watering of eyes. Stiffness of neck unchanged.

*Feb.* 12*th*. Very much less languid; better spirits. Very slight cough occasionally, more in evening; no concomitant symptoms; stiffness of neck gone (thus verifying Hahnemann's statement, that the oldest symptoms disappear *last* in a homœopathic permanent cure); all day a feeling of slight soreness of muscles of nape when pressing them, and slight stiffness of nape on bending head back (effect of *Nat. mur.?*) Bowels open twice yesterday and to-day, otherwise? natural.

13*th*. Cough troublesome only in evening, but without the former concomitants; bowels open twice.

14*th*. Cough slightly increased, with expectoration; he was out of doors last night in the chilly air. Slight soreness of left ala nasi, externally, worse on touching and blowing nose, with heat, redness and swelling; this he has had before, and he thinks yesterday; it is a marked symptom of *Nat. mur.* His expectoration has increased since he took the dose.

15*th*. Ala nasi better; no other symptoms.

26*th*. The usual fullness of head on waking has continued as before, up to to-day. For the last two or three days, a mental feeling, as if something was coming on (effect of *Nat. mur.?*) At 11 A. M., after breakfast, felt something wrong about right supra-orbital ridge, and found it was sore to touch, as before, but to a less extent; not much in evening. (He never had such neuralgia before this attack.)

Since he took the dose, bowels act every day; formerly they would sometimes not act for two days. Occasional momentary pains along both sides of neck, in a direction downward and forward, affecting the whole extent of their course simultaneously (effect of *Nat. mur.?*)

The neuralgia had returned. Was the dose to be repeated or allowed to act, or a new remedy given? Sometimes a dose will give instantaneous relief, followed by a return of the symptoms as severe as ever. In this case it is simply the palliative action of the medicine, and a new one should be selected. (See HAHNEMANN'S Preface to *Magnet*, and Ad. Lippe in *Hahnemannian Monthly*, vol. i, p. 372.) Sometimes, after the dose has afforded more gradual but complete relief, the symptoms will return in a modified or lessened form, after an interval of a few days. In these cases, it is simply an instructive proving of the medicine, which will disappear if left to itself. (See HAHNEMANN'S *Chronic Diseases*, and Ad. Lippe in *Hahnemannian Monthly*, vol. ii, p. 26.) No medicine is, therefore, given.

*27th.* About 10 A. M., after breakfast, pain as if right eye were pressed out; the eye waters, and the lid falls a little; the inner half of the right supra-orbital ridge and the corresponding part of forehead just above it are sore to touch and on *knitting the brows*, the soreness being in the bone; feels as if he must often draw the hand lightly over that part of the forehead, which relieves it. The pains prevent him from reading; they were worse than yesterday, but less than during the first attack; no soreness under right supra-orbital ridge. In afternoon, both before and after dinner, the symptoms were better, but again slightly increased by writing and book work, going off entirely in the course of the afternoon. Has a few pimples on forehead, one with a vesicular head; has these about every three weeks (effects of *Nat. mur.?*) No fullness of head on waking.

*28th.* About 10 A. M., after breakfast, the same symptoms as yesterday, but much less; lasting till afternoon; gradually decreasing.

*29th.* Between noon and 1 P. M., slight soreness along right eyebrow, in the bone, when pressed; gradually lessening; not felt in the evening.

*March 1st.* About 10 A. M., the same soreness as yesterday; felt slightly all day.

*6th.* The fullness of the head on waking returned. Several weeks afterward he reported that he had been quite free from neuralgia and the stiffness of neck on waking.

1869, *August.* Has had no return of neuralgia or stiffness of neck. No report given as to the fullness of head.

The patient went on with his avocations as usual, proving that the inhalation of crude drugs does not necessarily interfere with the action of high potencies.

N. B. After he was cured, he ridiculed the idea that one little globule could have had any effect on him. Subsequently, however, he applied to me for treatment for some other complaint; but I declined to prescribe, telling him that there were plenty of medicines in his shop, if he did not believe in what I had done for him. Ungrateful patients should be punished.

*Manganum Carb. in colic.*—1870 Mrs. ——— complained of intense pain, as if bowels were drawn together, beginning in stomach, going downwards to abdomen; chiefly on left side; it begins one and a quarter hour after food, and attains its highest two hours after food. The pain is relieved by bending double, and especially by sitting bent before a fire, also by food, and by eructations; worse in cold weather or a cold room; the pain concentrates itself around and above navel. This has lasted for more than three weeks. She has taken *Nux* with only temporary relief.

*Diagnosis of remedy.*—Constrictive pains about navel. *Acon., Anac., Asaf., Bell., Graph., Mag. mur., Mang., Natr., Phos. ac., Plumb., Plat., Ran. sc., Rhus, Sulph., Thuy., Verb.*

Abdominal symptoms relieved by bending double, *Bell., Coloc., Mang., Merc., Natr. m., Puls., Rhus, Sars.;* by warmth, *Alum., Amm. c., Canth., Coloc., Laur., Mang., Nux mos., Nux vom., Rhus, Sabad., Sil., Staph., Stront.;* by food, *Bov., Laur., Mag. c., Mang., Merc., Natr., Rhus, Sabad., Stann.;* by eructation, *Ambr., Ant. t., Carbo veg., Colch., Ign., Kali, Nit. ac., (Phos.), Rhod., Sep., Sil., Sulph., Zinc;* worse by cold, *Mang., Nux, Rhus, Sabad., Mang. and Rhus* are equally indicated so far. According to Hering's law of Inverse Directions—which is simply a corollary to, or extension of Hahnemann's own teaching—the remedy for this case should produce a contractive pain (or at least some kind of symptom) going *upward* from abdomen. *Rhus* has not this exact symptom; it has "griping in abdomen, with oppression, mounting upwards, while sitting;" while *Mang.* has "*constriction,* nausea and warmth rising from middle of abdomen into chest (pharym.") *Rhus* has "*contractive* pain in right side, extending toward stomach; whereas the pain in the above case was chiefly on the *left.* Lastly *Mang.* has "distressing sensation in stomach; *thought she would feel better if she could eructate.*" Accordingly one dose (two globules) of *Mang. Carb.* 200, was given. A week afterward, she reported that the pain ceased at once, and that she could now bear exposure to cold. There was no return.

*Comments.*—This case was worked out from an enlarged copy of *Bœnninghausen's Repertory,* and clearly shows the absolute necessity of a *collective* of the conditions belonging to *every* symptom of an organ, such as I have adopted in my own Eye Repertory. The provings of *Mang.* show "distressing sensation in stomach, ceasing after dinner," but not "contractive pain" relieved by the same; thus demonstrating that the conditions of one symptom often apply equally well to others; sometimes being perhaps of *universal* application. It affords also another illustration of the value of clinical symptoms to fill up the gaps in the pathogenesis of our remedies; many of the above conditions are not to be found *as yet* in the provings of *Mang.*, but have been discovered and added by Bœnninghausen.

*Spongia in Chronic Winter Cough.*—December 9th, 1868. Mr.

——, æt. 53, has had cough every year from the beginning of October to end of May, for the last fourteen years. Has taken for it Paregoric and other domestic medicines, but without relief; no medicine for the last twelve months.

*Present symptoms.*—Cough caused by tickling in throat; expectoration easy, sometimes tastes salt; cough is worse on rising from bed and when indoors; it is excited by smoking tobacco (which he does daily); by lying on back or right side, especially the former; by drinking milk, ale, spirits, *cold* tea, or *cold* water; relieved by eating, and by *warm* tea or *warm* coffee; it disturbs his sleep and that of his wife also; it is worse in wet weather, better in frosty weather.

*Diagnosis of the remedy.*—Those who alternate would be puzzled, as they would have to alternate at least *seven* medicines in order to cover all the symptoms of this case. Hahnemann's rule of covering the *totality* of the symptoms, cannot always be carried out, as in the present case; he has, therefore, to given us another rule, viz., to select the remedy chiefly, and first according to those symptoms which are *most strange, peculiar and characteristic.* Now the repertories give the following. [Note other medicines have been since added to the following list, but I give it just as it stood at the time, to show exactly how the case was worked out.]

Cough better by food, *Anac., Ferr., Spong.*

Cough better by warm drinks, *Ars., Lycop., Nux, Rhus., Verat.*

Cough worse when lying on right side, *Acon., Amm. m., Carbo. an., Ipec., Stann.*

Cough worse when lying on back, *Amm. m., Iod., Nux, Phos., Sil.*

Cough worse from cold drinks, *Amm. m., Calc., Carb. v., Dig., Hep., Lycop., Rhus, Scill., Sil., Staph., Sulph. ac., Verat.*

Cough worse from tobacco, *Acon., Brom., Bry., Carb. an., Clem., Coloc., Dros., Euphr., Ferr., Hell., Hep., Ign., Iod., Lach., Mgs. arct., Mag. c., Nux Petr., Puls., Spon., Staph., Sul. ac.*

Cough worse on rising from bed, *Bry., Carb. v., Cocc., Con., Lach.*

Cough worse in doors, *Arg., Bry., Croc., Laur., Magn. c., Magn. m., Nat. mur., puls., Spig.*

Cough worse from coffee, *Caps., Caust., Cham., Cocc., Ign., Nux.*

Cough worse from beer, *Meg., Rhus.*

Cough worse from milk, *Ant., Ant. t., Brom., Kali., Sul. ac., Zinc.*

Cough worse from tea, *Ferr.*

Salt Sputa. *Alum.*, *Ambra.*, *Amm. c.*, *Ant. t.*, *Arsen.*, *Baryt. c.*, *Bov.*, *Calc.*, *Cann.*, *Carb. v.*, *China*, *Cocc.*, *Con.*, (*Dros.*,) *Euphor.*, *Graph.*, *Hyos.*, *Iod.*, *Kalmia*, *Lach.*, *Lycop.*, *Magn. c.*, *Mang.*, *Merc.*, *Mez.*, *Nat. c.*, *Nat. mur.*, *Nit. ac.*, *Phos.*, *Puls.*, *Rhus.*, *Samb.*, *Sep.*, *Spong.*, *Stann.*, *Sulph.*, *Sul. ac.*, *Verat.*

The most *peculiar* and *characteristic* symptoms seemed to be the relief of the cough from eating, which occurs only under *three* medicines, *Anac.*, *Ferr.* and *Spong.* Of these, *Anac.* covers one symptom only; *Ferr.* and *Spong*, cover three; thus the choice is reduced to these two. Both *Ferr.* and *Spong.* have cough relieved by eating, and aggravated by tobacco; *Ferr.* has aggravation from tea; *Spong.* has salt sputa. Which of these symptoms is to decide? If aggravation from tea means *tea* as such, then *Ferr.* is contra-indicated, inasmuch as tea (if warm) relieved. If it means aggravation from *warm* tea, such as is usually taken, it is contra-indicated still more. On the other hand, *Spong.* is also contra-indicated by the relief from drinking; this, however, was not so strong a contra-indication, as the patient's cough was relieved by some drinks. I therefore gave him one dose (a globule) of *Spong.* 2000th (Jenichen), on December 9th.

December 18th. Cough better altogether, especially during the day; sputa easier, do not taste salt; less tickling in throat before cough; smoking does not now excite cough as much, and he can lie better on back or right side; sleeps better, and feels much better generally. *Has made no change whatever in diet or regimen.*

1869, January 19th. Reports that for the past fifteen days has been quite well in every respect; appetite much better than formerly; can smoke without inconvenience; says he has not felt so well for fourteen years, though it is now wet weather.

1869, December. Still has remained quite well.

Thus chronic winter cough, of fourteen years' duration, was cured in less than a month, by a single globule of a very high potency of the *homœopathic* remedy.

It may be asked, why the symptoms "worse from beer," were not more characteristic than "relief from food," as only *two* remedies have it; or "worse from tea," which belongs only to *one.* The reason was that the aggravation was not from beer or tea only, but from *cold* drinks; hence I consider it due, not to the beer or tea themselves, but to the *coldness* of the drinks. Had the cough been increased by beer or tea only, and not by other cold drinks, it would have been a different matter.

The symptoms "cough worse by lying on right side or back, better by eating," I afterwards verified in another case.

*Carbo. veg., in cough.*

September 1st, 1869. Mrs. ——, caught cold fourteen days ago; has had cough for a week, which allopathy has failed to relieve; the cough is worse by day, in open air and during supper; it is continuous, hard and dry; soreness of chest and heat of body when coughing; feeling of mucus in throat at night, choking her when she coughs, the choking is relieved when sitting up or moving; itching (internally) from throat down centre of chest; worse when coughing.

*Diagnosis of remedy:*

Itching in chest, *Agar., Ambr., Carb. veg., Kali, Meny., Mez., Phos., Phos. ac., Spig.*

Breathing relieved by sitting up, *Ant. t., Asaf., Carb. v., Cham., Ferr., Lyc., Phos., Puls., Rhus., Samb.*

Breathing relieved by moving, *Rhus, Samb.*

Choking with cough, *Acon., Ant., Bry., Hep., Ipec., Led., Mgs. Mgs. Arct., Oleand., Puls., Rhus.*

Cough from eating, *Anac., Ant. t., Arsen., Baryt. c., Bell., Bry., Calc., Caust., Carb. v., Cham., Chin., Cocc., Cap., Dig., Ferr., Hep., Hyos., Ipec., Kali., Laur., Lycop., Mag. c., Mag. m., Mez., Mosch., Nux, Op., Phos., Puls., Rhus., Ruta., Sep., Sil., Staph., Sulph., Verat., Zinc.*

Cough in open air, *Arsen., Baryt. c., Bry., Calc., Carb. v., Cham., Cina., Cocc., Dig., Ipec., Lycop., Mgs. arct., Mosch., Nit. ac., Nux, Phos., Rhus., Seneg., Sil., Spig., Staph., Sul. ac.*

*Phos.* and *Carb. veg.* so far were equally indicated; both have hard, dry cough and soreness of chest; *Carb. veg.*, alone, has heat when coughing. One dose (a globule) of *Carb. veg.* 3,000 (Jenichen), was given; no change in diet, etc.

September 18th. Reports that improvement commenced in a few hours; mucus looser the same night; next day almost gone. Her husband says that she has not been so well for seven years. There was no change of weather to account for this improvement.

*Gamboge in diarrhœa.*

1870, April 18th. A boy aet. fourteen months, has had diarrhœa for four days, the weather being hot; stools copious, watery yellow, or like curdled milk, of offensive, sickening smell, expelled with much force; has had three stools this morning; vomits food undigested. His father, who does not believe in homœopathy, has given him some allopathic medicine, without relief.

*Diagnosis of remedy* (according to Bell's most excellent Repertory).

Stools watery yellow, *Apis*, *Arsen.*, *Borax*, *Canth.*, *Chin.*, *Crot.*, *Dulc.*, *Gamb.*, *Grat.*, *Hyos.*, *Thuy.*

Stools copious, *Ant. c.*, *Ant. t.*, *Benz. ac.*, *Cact.*, *Colch.*, *Cod.*, *Cub.*, *Dios.*, *Elat.*, *Gamb.*, *Iris*, *Jatro.*, *Iod.*, *Kali. bichr.*, *Lept.*, *Mag. c.*, *Nux mos.*, *Pod.*, *Raph.*, *Rumex*, *Sec.*, *Thuy.*, *Verat.*

Stools like curdled milk (not recorded).

Stools offensive, *Apis*, *Arsen.*, *Benz. ac.*, *Coff.*, *Corn.*, *Gamb.*, *Graph.*, *Lact.*, *Lith. ac.*, *Mez.*, *Nux.*, *Op.*, *Psor.*, *Puls.*, *Rumex.*, *Scill.*, *Sec.*, *Sulph. ac.*

Stools forcible, *Cist.*, *Crot.*, *Gamb.*, *Grat.*, *Jatr.*, *Kali. bichr.*, *Nat. c.*, *Nicc.*, *Phos.*, *Rhad.*, *Rhod.*, *Sep.*, *Sulph.*, *Thuy.*

Thus *Gamboge*, alone, corresponds to these symptoms, one dose of which was given at once, in the 200th dilution.

The child had one diarrhœic stool soon after the dose, subsequent to which, there was no return of the symptoms. His father now believes that there *is* something in homœopathy. For the symptoms of *Gamboge*, we are chiefly indebted to Nenning, whose provings have been ridiculed by mongrels as "unreliable," "eminently misleading," "made to order," etc., etc., etc. His symptoms, however, have proved *perfectly* reliable in the hands of those who had sufficient intelligence and industry to practice homœopathically, and of late some of the mongrels have had to eat their own words on this subject.

